# Role of topology in the spontaneous cortical activity

**DOI:** 10.1186/1471-2202-16-S1-P6

**Published:** 2015-12-18

**Authors:** Silvia Scarpetta, Antonio de Candia, Ilenia Apicella

**Affiliations:** 1 Department of Physics "E.R.Caianiello" & INFN, University of Salerno, Fisciano,84084, Italy; 2 Department of Physics, University of Napoli "Federico II", Napoli, Italy; 3 INFN Sezione di Napoli, Napoli, Italy

## 

**Figure 1:**
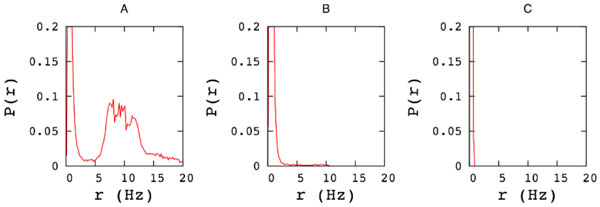
**Distribution of the spiking rates for the model with (A) no reshuffling *f *= 0, (B) reshuffling a fraction *f *= 0.1 of connections, and (C) all connections reshuffled *f *= 1**. All other parameters are the same (N = 3000 neurons, *H*_0_/Θ = 0.221, noise variance α = 0.06 ms^−1^) in the three cases.

Spontaneous cortical activity can show very complex collective emerging features, with, in some cases, the alternation of "down states" of network quiescence and "up states" of generalized spiking and neuronal depolarization [1]. Results on in vitro and in vivo up states has suggested that this spontaneous activity occurs in a highly structured way, with repeating spatiotemporal patterns of cellular activity [1,2]. Because of their stereotyped spatio-temporal dynamics, it has been conjectured that network up states are circuit attractors that could implement memory states [1]. The precise mechanisms by which these up states transitions occur is still unclear, but it seems to rely on network mechanisms [1]. Previous papers [3,4] have studied a leaky IF model whose connectivity was designed in such a manner as to favor the spontaneous emergence of collective oscillatory spatio-temporal patterns of spikes. In this model the alternation of up and down states does not depend on a kind of neuron bistability, nor on synaptic depression, but is rather a network effect. The model consists in N leaky IF units, with Poissonian noise. Structured connections Jij was fixed with a "learning procedure" [3,4] inspired to spike time dependent plasticity (STDP). After the learning and pruning procedure [3], about 12% of the N(N-1) connections survive as positive connections, and about 27% as negative connections. There's a balance condition, such that the sum of connections entering on a single neuron ΣjJij is of order 1/√N, and therefore vanishes in the thermodynamic limit. In order to check if the transition region with bimodal distribution of the firing rate survive even after changes in the topology of the network, in this work we reshuffle a fraction of the connections. For each chosen connection we change the presynaptic neuron, choosing as the new presynaptic neuron a random neuron of the network. After reshuffling a fraction f of connections, not only the number of excitatory and inhibitory connections is the same as before, but also the strengths of the connections are the same, and only the topology (number of loops, mean path length, clustering coefficient, etc.) is changed. We observe that reshuffling the connections changes the features of the dynamics dramatically. Before reshuffling, i.e. with f = 0 and connections dictated by the learning rule of [3], the system has a transition from a regime of Poissonian noise activity to a regime of spontaneous persistent collective replay, and at the transition point the network dynamics shows an intermittent reactivation of the stored patterns, with alternation of up and down state, and bimodal distribution of spiking rate (fig. 1A). Reshuffling a small fraction of connections, leaving all the other parameters unchanged, we observe that the transition region with bimodal distribution disappears, and the dynamics is Poissonian with unimodal rate distribution for all the parameters investigated. These results shows the role of topology in dictating the emerging collective dynamics of neural circuits.
